# Intranasal Administration of Oxytocin Attenuates Social Recognition Deficits and Increases Prefrontal Cortex Inhibitory Postsynaptic Currents following Traumatic Brain Injury

**DOI:** 10.1523/ENEURO.0061-21.2021

**Published:** 2021-06-10

**Authors:** Avery Runyan, Dana Lengel, Jimmy W. Huh, Jessica R. Barson, Ramesh Raghupathi

**Affiliations:** 1 Program in Neuroscience, Graduate School of Biomedical Science and Professional Studies, Drexel University College of Medicine, Philadelphia, PA 19129; 2 Department of Neurobiology and Anatomy, Drexel University College of Medicine, Philadelphia, PA 19129; 3 Department of Anesthesiology and Critical Care Medicine, Children’s Hospital of Philadelphia, Philadelphia, PA 19104

**Keywords:** excitability, GABAergic neurotransmission, intranasal administration, oxytocin, pediatric TBI, social behavior

## Abstract

Pediatric traumatic brain injury (TBI) results in heightened risk for social deficits that can emerge during adolescence and adulthood. A moderate TBI in male and female rats on postnatal day 11 (equivalent to children below the age of 4) resulted in impairments in social novelty recognition, defined as the preference for interacting with a novel rat compared with a familiar rat, but not sociability, defined as the preference for interacting with a rat compared with an object in the three-chamber test when tested at four weeks (adolescence) and eight weeks (adulthood) postinjury. The deficits in social recognition were not accompanied by deficits in novel object recognition memory and were associated with a decrease in the frequency of spontaneous inhibitory postsynaptic currents (IPSCs) recorded from pyramidal neurons within Layer II/III of the medial prefrontal cortex (mPFC). Whereas TBI did not affect the expression of oxytocin (OXT) or the OXT receptor (OXTR) mRNAs in the hypothalamus and mPFC, respectively, intranasal administration of OXT before behavioral testing was found to reduce impairments in social novelty recognition and increase IPSC frequency in the mPFC in brain-injured animals. These results suggest that TBI-induced deficits in social behavior may be linked to increased excitability of neurons in the mPFC and suggests that the regulation of GABAergic neurotransmission in this region as a potential mechanism underlying these deficits.

## Significance Statement

Traumatic brain injury (TBI) occurs in approximately half a million children below the age of 14 each year, with children younger than four years old at heightened risk. A younger age at injury is associated with worse behavioral and psychosocial outcomes in pediatric TBI patients, particularly as children age into adolescence and adulthood. In this study, we explored the role of oxytocin (OXT) in the long-term effects of pediatric TBI on social behaviors in adolescence and adulthood. The results indicate that intranasal administration of OXT improves social outcomes following pediatric TBI, potentially by increasing inhibitory neurotransmission within the medial prefrontal cortex (mPFC) and provide novel support for the use of intranasal OXT treatment to mitigate social deficits in pediatric TBI patients.

## Introduction

Nearly half a million children younger than 14 years old suffer from a traumatic brain injury (TBI) each year (Faul and Coronado, 2015). Pediatric TBI is associated with poor psychosocial outcomes in adolescence and young adulthood (Levin et al., 2004; Anderson et al., 2012; Rosema et al., 2012; Ryan et al., 2016), such as lower scores in communication, emotional perception, social skills, and fewer relationships (Ryan et al., 2014, 2016; Douglas, 2020). Chronic social and behavioral difficulties are the most prevalent and disabling outcome of pediatric TBI patients (Zamani et al., 2020), although these psychosocial deficits have historically received less attention in preclinical pediatric TBI studies.

A few preclinical studies to date have investigated social behavior as an outcome after pediatric TBI. Closed head injury in juvenile (21-d-old) mice led to impaired social recognition using the three-chamber test at four weeks postinjury (adolescence) and deficits in sociability at eight weeks following injury (adulthood; Semple et al., 2012, 2017). Contusive brain trauma in neonate (14-d-old) rats resulted in both sociability and social recognition deficits two weeks following injury (Wei et al., 2016). The mechanisms underlying these social deficits following pediatric TBI are not fully understood. TBI in 21-d-old mice resulted in diminished neuronal arbor complexity within the medial prefrontal cortex (mPFC) at eight weeks postinjury (Semple et al., 2017), a region that has been implicated in social processing in both rodents and humans (Ko, 2017).

Oxytocin (OXT), a neuropeptide known to affect PFC function and social behavior (Gimpl and Fahrenholz, 2001), is involved in social bonding and trust (Tops et al., 2013). Administration of OXT is reported to improve social recognition deficits in mouse models of autism (Andari et al., 2010; Sala et al., 2011; Hara et al., 2017). Transplantation of hypoxia-conditioned induced pluripotent stem cell-derived progenitor cells shortly after neonate TBI in rats improved sociability and social recognition, which was associated with an increase in both OXT and OXT receptor (OXTR) levels in the injured cortex (Wei et al., 2016).

The majority of central nervous system OXT is produced within the paraventricular nucleus (PVN) in the hypothalamus (Knobloch et al., 2012), from which OXT producing neurons project to various brain regions including the mPFC (Bakos et al., 2018; Jurek and Neumann, 2018). Neuronally expressed OXTRs are G-protein-coupled receptors which are typically coupled to G_αq_ and activate downstream signaling pathways involving protein kinase C (Bakos et al., 2018). Within the mPFC, OXTRs are predominantly expressed on inhibitory somatostatin (SOM) neurons and have been implicated in the modulation of social behaviors (Nakajima et al., 2014). OXT has been found to increase spontaneous inhibitory postsynaptic currents (IPSCs) and the release of GABA (Wrobel et al., 2010; Harden and Frazier, 2016). We previously demonstrated that TBI in 11-d-old rats results in an increase in spontaneous EPSCs and a concomitant decrease in spontaneous IPSCs in Layer II/III pyramidal neurons within the mPFC (Lengel et al., 2020). Increasing excitatory currents in the PFC decreases social exploration in rodents (Yizhar et al., 2011; Bicks et al., 2015), suggesting that changes in excitation/inhibition balance within the medial PFC may be a mechanism underlying deficits in social behaviors following TBI.

The purpose of the current study was to define the extent of social behavior deficits following a moderate closed-head injury in the 11-d-old rat and to determine whether OXT treatment would reverse these deficits. To investigate whether the behavioral effects of OXT may be facilitated by its actions within the medial PFC, we measured the effects of bath application of OXT on electrophysiological properties of medial PFC pyramidal neurons using whole-cell patch clamp recordings.

## Materials and Methods

### Animals

All animal procedures were performed in accordance with the regulations of the Institutional Animal Care and Use Committee and followed the National Institutes of Health *Guide for the Care and Use of Laboratory Animals*. Timed-pregnant [embryonic day (E)20] female Sprague Dawley rats were purchased from Charles River Laboratories and gave birth in the animal facility (average litter size was between 11 and 13 pups). A caveat to the present study is that the stress of transportation may affect social behavior in the offspring. Animals were housed under a normal 12/12 h light/dark cycle (lights on from 7 A.M. to 7 P.M.) with *ad libitum* access to standard rat chow and water. Age-matched male and female rats used as the familiar and novel rats in the three-chamber test were also purchased from Charles River and housed in the same room under the same conditions as the test subjects. The pups were weaned on postnatal day 21 and group housed under the same conditions. Animals were handled for 5–10 min at least two to three times per week before the start of behavioral assessment.

### TBI

The model of moderate pediatric TBI used in this study was originally characterized by Raghupathi and Huh (2007) and was subsequently used in multiple studies (Hanlon et al., 2017, 2019; Lengel et al., 2020). Animals from each litter were randomly assigned to sham-injury or brain-injury groups. Sham (*n* = 52) or brain injury (*n* = 60) was administered on postnatal day 11, the neurologic equivalent of a child below the age of 4 (Porterfield, 1994; Yager and Thornhill, 1997; Rice and Barone, 2000). Male and female rat pups were anesthetized using isoflurane (Patterson Veterinary, 5% induction, 2–3% maintenance) and an incision was made to expose the skull. Animals were then placed in a plastic rodent restrainer (Braintree Scientific Braintree MA) and moved onto the stage of an electronic controlled cortical impact (eCCI) device (Custom Design International, Richmond VA). The impactor tip was driven into the intact skull at a velocity of 5 m/s (3-mm distance from zero point, 100-ms dwell time) over the left lateral hemisphere midway between the bregma and lambda. After impact, the pup was placed in a supine position and the time until the pup righted itself onto all four paws was measured. After righting, pups were then placed back on isoflurane and examined for hematoma and skull fractures, and the incision was sutured closed. Sham-injured animals were surgically prepared but did not receive an impact. The total time under anesthesia for brain-injured and sham-injured animals did not exceed 10 min. Animals were placed in a separate cage to recover and were subsequently returned to the dam. Surgical procedures and recovery were performed on a heating pad to maintain the body temperature at 37°C.

### Behavioral testing

All behavioral tests were conducted in the dark under red light with the three-chamber test being done first at four or eight weeks postinjury, followed by novel object recognition memory test, and assessment of locomotor activity (week 5 for the OXT treatment arm of the study). All behaviors were recorded and testing and scoring from videos were performed by an evaluator who was blinded to the injury and treatment status of the animals.

### Three-chamber test

Social behavior was quantified using a three-chamber test. Rats were tested four or eight weeks after injury. The three-chamber apparatus was custom made, comprised of three chambers with two Plexiglas dividers with a 10-cm opening to allow the rat to move between the two far chambers (40 × 40 cm) and a middle chamber (20 × 40 cm). A camera equipped with an infrared detector was used to record the behavior of the test rat in each of the stages, from which the time spent sniffing the rat/cup (stage 2), or the novel/familiar rat (stage 3), was determined. In the first stage, the rat was habituated to the three-chamber apparatus in the dark for 5 min, following which it was herded into the middle chamber and the Plexiglas doors were lowered to keep the animal in the middle chamber. In preparation for stage 2, an age-matched and sex-matched rat that the test rat had never seen before was put in an inverted wire mesh cup (14 cm in diameter, 20 cm in height) in one of the outer chambers and an identical empty cup was placed in the opposite chamber. Stage 2, which measures sociability, began once the Plexiglas doors were raised. This stage lasted 10 min, and the number of seconds the test rat spent sniffing the “rat cup” and “empty cup” was counted. At the end of this stage, the lights were turned on and the test rat was again herded back into the middle chamber and the Plexiglas doors closed. Another new novel rat was placed in the empty cup. In stage 3, social recognition was measured as the time the test rat spent sniffing the “familiar rat” (from stage 2) and the “novel rat” over 10 min. The discrimination index (DI) in stage 2 used the equation [(time sniffing rat cup) – (time sniffing empty cup)]/[(time sniffing rat cup) + (time sniffing empty cup)] and, for stage 3, the equation [(time sniffing novel rat) – (time sniffing familiar rat)]/[(time sniffing novel rat) + (time sniffing familiar rat)].

### Novel object recognition

To assess non-social memory, a novel object recognition paradigm was used. All testing and habituation occurred in the dark and the behaviors were recorded using a camera equipped with an infrared detector. Rats were first habituated over 2 d to a plastic box (61 × 41 cm) for 10 min each day. On the third day, rats began the novel object recognition test by being familiarized with two identical objects placed in opposite corners of the box for 5 min. Rats were returned to their home cage, then returned 1 h later to the box with one of the objects switched for a new (“novel”) object. The time the rat sniffed the “familiar” object versus the novel object was determined. DI was calculated using the following equation: [(time sniffing novel object) – (time sniffing familiar object)]/[(time sniffing novel object) + (time sniffing familiar object)].

### Locomotor activity

To assess any motor deficits, rats were tested individually in 43.2 × 43.2 cm activity monitor boxes (Activity Monitor version 5, Med Associates) during a 30-min period. The total distance traveled, as tracked by the number of beam breaks, was measured and averaged in 5-min bins for each animal.

### Quantitative real-time PCR

At the conclusion of behavioral testing in adolescence (five weeks postinjury), tissue from the PVN and PFC regions of a subset of rats (11 sham, 13 injured) were dissected after decapitation of male and female rats for quantitative real-time PCR (qRT-PCR). For dissection of the PVN, coronal slices were obtained between −1.2 and −2.2 mm from bregma. For dissection of the PFC, coronal slices were obtained between +3.0 and +4.0 from bregma. The PVN was dissected as a reversed isosceles triangle, 1.0 mm bilateral to the third ventricle and between the fornix structures. The PFC was dissected in a diamond shape, bilateral from the dorsomedial tip of the slice to the corpus callosum, and then along the border of the corpus callosum to its ventral tip. Tissue was then stored in RNA Later (QIAGEN Inc.) at −20°C until further processing. The RNA from the tissue was extracted using a RNeasy Mini kit (QIAGEN Inc.) along with DNase 1 (QIAGEN Inc.). RNA yields were measured with a NanoDrop Lite spectrophotometer (Thermo Electron North America LLC) and resulted in A_260_/A_280_ ratios of 2.0–2.1, indicating high purity. RNA was converted to cDNA using SuperScript VILO Master Mix (Invitrogen) in a SimpliAmp Thermal Cycler (Applied Biosystems). Triplicate samples of cDNA, SYBR Green PCR reagent (Applied Biosystems), and target primer or cyclophilin primer were run on 96-Well MicroAmp Fast Optical Reaction Plates (Applied Biosystems). The protocol was set to be 2 min at 50°C, 10 min at 95°C, 40 cycles of 15 s at 95°C, and 1 min at 60°C. Primers were designed with the help of the NCBI Primer design tool (http://www.ncbi.nlm.nih.gov/tools/primer-blast/) and purchased from Invitrogen at ThermoFisher Scientific. Serial dilutions of primers were tested for specificity and efficiency on sham tissue of the respective regions. Only primers with one peak in the melt curve indicating good specificity were used. Efficiency was determined by serial dilutions of primers and graphing log of the dilutions against the threshold cycle (Ct) values. Target mRNA expression was quantified relative to cyclophilin-A using the relative quantification method (ΔΔC_T_), which was quantified using the following equation: −2^(Average CT values of target gene-Average CT values of Cyclophilin-A)^. We chose to use cyclophilin-A as a reference gene in this study because it was reported to be a stable housekeeping gene in multiple brain injury paradigms (Langnaese et al., 2008; Swijsen et al., 2012; Timaru-Kast et al., 2015). The sequences for the primers were as follows: cyclophilin-A (cyc; 200 nm), forward: 5′-GTGTTCTTCGACATCACGGCT-3′ and reverse: 5′-CTGTCTTTGGAACTTTGTCTGCA-3′; OXT (200 nm), forward: 5′-ATCTGCTGTAGCCCGGATGG-3′ and reverse: 5′-GAAGGAAGCGCCCTAAAGGT-3′; OXTR (100 nm), forward: 5′-GGGCCACCACAACGCAACGAG-3′ and reverse: 5′-AGACCGCCCAGCAATCGAAG-3′.

### Intranasal OXT administration

The following intranasal administration protocol was adapted from (Brabazon et al., 2017) and (Meidahl et al., 2018). Six days before any behavioral intranasal OXT testing, rats began a protocol to familiarize them to receiving intranasal drops. On the first day, rats were placed into a DecapiCone disposable rodent restrainer (Braintree Scientific) with the end sealed off but which allowed the rat to move relatively freely in the bag for 10 min. The next day, the rats were restrained in the nose cone and flipped on their back for 1 min. From the third to fifth day approximately three drops of saline were intranasally given to each rat. To administer drops, rats were restrained in the DecapiCone and flipped on their back. Drops of 6 μl of saline were pipetted using a micropipette into each naris. After the last drop, rats remained on their back for another minute to ensure the saline or OXT did not drip out. Animals that sneezed out all the drops were given additional drops to better acclimate them. On the day of testing, rats were given OXT (O4375, Sigma-Aldrich) or saline 1 h before testing. Sham-injured and brain-injured rats were randomly assigned to receive either 20 or 60 μg of OXT (Hara et al., 2017; Meidahl et al., 2018) or saline.

### Slice preparation

**Figure 1.**
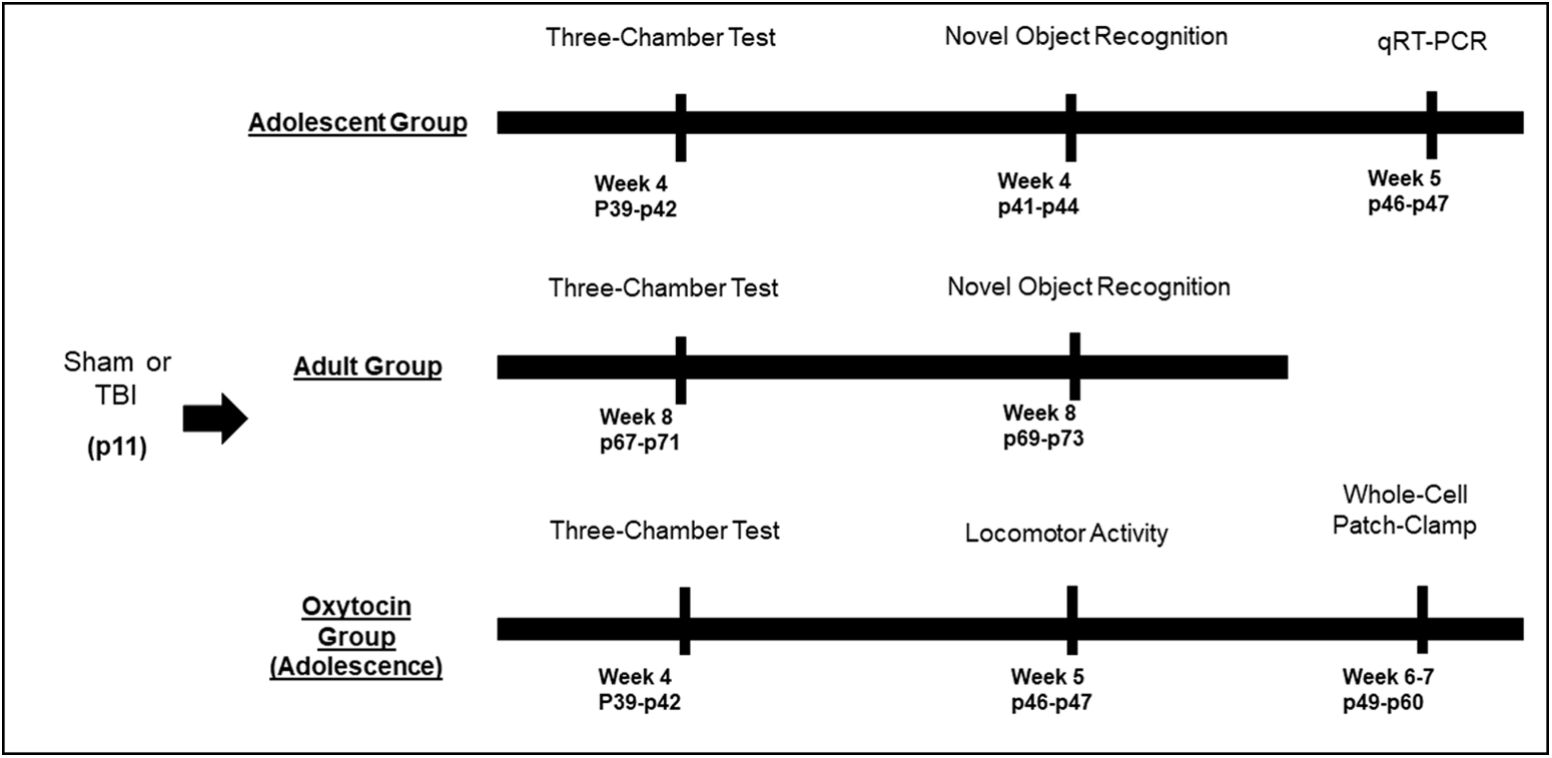
Timeline of experiments. Eleven-day-old rat pups were subjected to TB1 or sham injury. Behavioral experiments were conducted in three separate cohorts of animals beginning at either four weeks postinjury (adolescent group and OXT group) or eight weeks postinjury (adult group). At the conclusion of the behavioral testing in adolescence, animals were used to generate tissue for qRT-PCR experiments, and animals from the OXT group were used for electrophysiological experiments.

All patch clamp recordings were done at the conclusion of behavioral testing in adolescence (Fig. 1). Rats were anesthetized with Euthasol (100 mg/kg, Patterson Veterinary) and then transcardially perfused with 60 ml of oxygenated slicing artificial CSF (aCSF) containing 34 mm sucrose, 11 mm glucose, 24 mm NaHCO_3_, 2.5 mm KCl, 1.25 mm NaH_2_PO_4_, 10 mm MgSO_4_, and 0.5 mm CaCl_2_ at pH 7.4. Brains were quickly removed and glued to the slicing stage of a vibrating microtome (Leica Microsystems), and 300 μm coronal slices containing the mPFC were cut between 3 and 4 mm anterior to bregma. Slices were incubated for 1 h at 37°C in oxygenated aCSF containing 126 mm NaCl, 10 mm glucose, 26 mm NaHCO_3_, 2.5 mm KCl, 1.25 mm NaH_2_PO_4_, 1 mm MgSO_4_, and 2 mm CaCl_2_ at pH 7.4. After incubation, the slices were allowed to equilibrate at room temperature for at least 1 h before recording.

### Whole-cell patch clamp electrophysiology

Brain slices containing the medial PFC were individually transferred to a recording chamber and continually perfused with oxygenated aCSF maintained at 34°C. In a subset of recordings, OXT was added to the bath solution at a 1 μm concentration, as previously used (Harony-Nicolas et al., 2017). OXT was bathed onto a slice for 10 min before the beginning of recording and was maintained for the duration of recording (three to five cells per slice). Using an Olympus BX51WI microscope and Samsung SCB-2001 camera, individual Layer II/III pyramidal neurons were identified with infrared differential interference contrast imaging. Borosilicate glass patch pipettes were pulled to a resistance of 5–8 MΩ and filled with 128 mm K gluconate, 10 mm HEPES, 0.05 mm CaCl_2_, 0.3 mm GTP, 5 mm ATP, 1 mm glucose, and 4 mm NaCl at pH 7.4 for whole-cell patch-clamp recordings of intrinsic excitability measures and for a subset of recordings of excitatory and inhibitory currents (*n* = 18 sham cells and 23 injured cells). Additional whole-cell patch-clamp recordings of excitatory and inhibitory currents (*n* = 14 sham cells and 21 injured cells) were obtained using an intracellular solution was used consisting of the following: 110 mm Cs-Gluconate, 10 mm CsCl, 1 mm EGTA, 1 mm CaCl_2_, 10 mm HEPES, and 1 mm HEPES, and adjusted to pH 7.3. Whole-cell recordings were acquired using an axon MultiClamp 700A amplifier and PClamp 9.2 data acquisition software (Molecular Devices), digitized using a DigiData 1332A digitizer (Molecular Devices) at 10 kHz, and low-pass filtered at 1 kHz. The access resistance was continuously monitored during recordings, and the recording was stopped if it exceeded 25 MΩ. A. Only neurons with a membrane potential of at least −60 mV and an action potential (AP) overshoot >0 mV were used in the analysis. If a neuron exhibited a non-accommodating, high-frequency spiking pattern in response to depolarizing current injections, it was deemed a fast-spiking interneuron and was excluded from further analysis. In voltage clamp mode, neurons were held at −70 mV to record spontaneous EPSCs for 5 min, and then at 0 mV to record spontaneous IPSCs, the reversal potentials for chloride ions and cations, respectively.

### Measurement of intrinsic excitability and synaptic properties

The data were analyzed using ClampFit 10.5 (Molecular Devices). The resting membrane potential was measured as the average voltage immediately after whole-cell configuration was achieved. The input resistance, rheobase, AP threshold, and AP amplitude were measured from current clamp recordings, which consisted of a series of depolarizing current steps (duration = 1 s) from −100 to 220 pA in 20-pA increments. Input resistance was determined from voltage responses to the first four hyperpolarizing steps (−100 to −80 pA). The rheobase current was determined as the minimal current needed to induce an AP. The AP threshold was measured as the voltage at the onset of an AP. The AP amplitude was measured as the difference between the threshold and the peak of an AP. In voltage clamp traces the spontaneous EPSCs and spontaneous IPSCs were analyzed using an automated template-matching protocol. The mean spontaneous current frequency was calculated for each cell across the full duration of the recording.

### Statistical analysis

Statistical analyses were performed using Statistica version 7.0 (StatSoft). All datasets were confirmed to contain a normal distribution and homogenous variances, as indicated by a Shapiro–Wilk and Levene’s test, respectively. An independent samples t test was used for comparisons between two means. For comparisons between more than two means, an ANOVA was used. *Post hoc* tests, when necessary were conducted using the Neuman–Keuls correction; *p* < 0.05 was considered significant. Kolmogorov–Smirnov statistical analysis was used to quantify differences among distributions (cumulative probability plots), in which case a *p* < 0.005 was accepted as significant.

## Results

### Acute response to injury

All brain-injured animals exhibited a skull fracture and hematoma immediately following injury on postnatal day 11 (data not shown). Brain-injured animals exhibited an increase in the time to right themselves following the impact (injury; *F*_(1,108)_ = 57, *p* = 0.000, ANOVA; Table 1), which did not differ between male and female rats (sex: *F*_(1,108)_ = 0.0005, *p* = 0.98, ANOVA). Brain injury also caused a brief period of apnea which did not differ between male and female rats (sex: *t*_(108)_ = 0.14, *p* = 0.88, unpaired *t* test). The latency of the righting reflex was similar in animals receiving saline, 20 μg OXT, and 60 μg OXT, confirming the randomization of animals to treatment groups (Table 1; treatment: *F*_(2,39)_ = 0.19, *p* = 0.82, ANOVA).

**Table 1:** Acute neurologic status of rats in the study

Outcome	Group	*N*	Righting reflex (s)	Apnea (s)
Behavior (adolescence)	Sham	16	69 ± 9	NA
	Injured	20	284 ± 40*	10 ± 2
	Behavior sham	12	70 ± 11	NA
	(Adult) injured	12	151 ± 16*	13 ± 1
Behavior (OXT)	Sham + saline	9	97 ± 24	NA
	Sham + OXT 1×	7	119 ± 22	NA
	Sham + OXT 3×	8	79 ± 15	NA
	Injured + saline	10	301 ± 32*	5 ± 1
	Injured+OXT 1×	7	306 ± 99*	6 ± 3
	Injured+OXT 3×	10	320 ± 50*	16 ± 4*

Eleven-day-old male and female rat pups were randomly assigned to either sham-injured or brain-injured groups. Sham-injured and brain-injured rats were randomly assigned to receive intranasal administration of 20 μg (1×) or 60 μg (3×) OXT. Subsets of the animals tested in the behavioral assays were randomly assigned to be euthanized for mRNA measurements and whole-cell patch clamp electrophysiology. Latency to regain righting reflex and times of apnea were recorded as described in Materials and Methods; *p < 0.05 compared with sham-injured rats.

### Effects of pediatric TBI on social behaviors during adolescence

**Figure 2.**
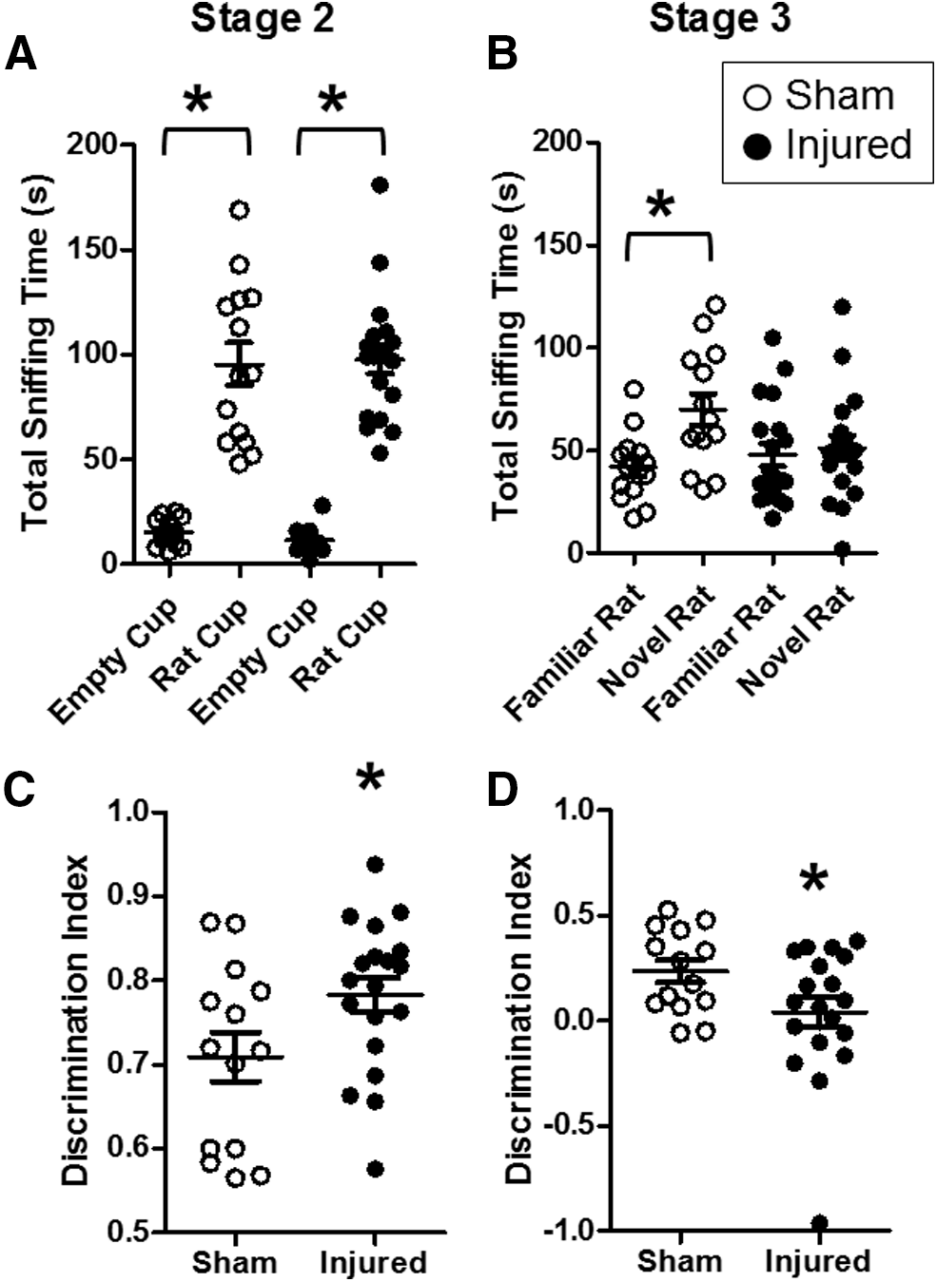
Pediatric TB1 impaired social recognition but not sociability during adolescence. Eleven-day-old male and female rat pups were tested for sociability (stage 2; ***A***, ***C***) and social recognition (stage 3; ***B***, ***D***) at four weeks after injury (adolescent age) using the three-chamber test as described in Materials and Methods. Data are presented as time (in seconds) spent interacting with rat/object (***A***, ***B***) or as DI (***C***, ***D***). Open symbols represent sham animals, closed symbols represent injured animals. Bars represent group mean values, and the error bars represent SEM; *p < 0.05.

Brain injury on postnatal day 11 did not result in impairments in sociability (stage 2) in adolescence (Fig. 2*A*,*C*). Both sham-injured and brain-injured animals spent significantly more time interacting with the rat cup compared with an empty cup, as measured by total sniffing time (*F*_(1,58)_ = 191.8, *p* = 0.00, ANOVA; Fig. 2*A*). The discrimination ratio during stage 2 was slightly but significantly higher in brain-injured rats compared with their sham-injured counterparts (*F*_(1,29)_ = 4.6, *p* = 0.04, ANOVA; Fig. 2*C*) although the total time spent sniffing both stimuli during stage 2 did not differ between sham and injured rats (data not shown; *F*_(1,29)_ = 0.18, *p* = 0.6, ANOVA). Brain-injured animals exhibited an impairment in social novelty recognition (stage 3) during adolescence (Fig. 2*B*,*D*). Statistical analysis of sniffing time during stage 3 revealed a significant interaction between injury status and stimulus (novel vs familiar; *F*_(1,58)_ = 6.3, *p* = 0.01; Fig. 2*B*). *Post hoc* tests showed that sham-injured animals spent significantly more time sniffing the novel rat compared with the familiar rat (*p* = 0.005) and compared with their brain-injured counterparts (*p* = 0.02). In contrast, brain-injured rats did not exhibit a significant difference in the time sniffing a novel rat compared with a familiar rat (*p* = 0.6). Statistical analysis also revealed that the DI during stage 3 was significantly lower in brain-injured animals compared with sham-injured animals (*F*_(1,29)_ = 4.75, *p* = 0.03, ANOVA; Fig. 2*D*). The total time spent sniffing both stimuli during stage 3 did not differ between sham and injured rats (data not shown; *F*_(1,29)_ = 2.4, *p* = 0.12, ANOVA). Overall, these experiments demonstrate that brain-injured male and female rats exhibit impairments in social recognition but intact sociability at four weeks postinjury.

### Effects of pediatric TBI on social behaviors during adulthood

**Figure 3.**
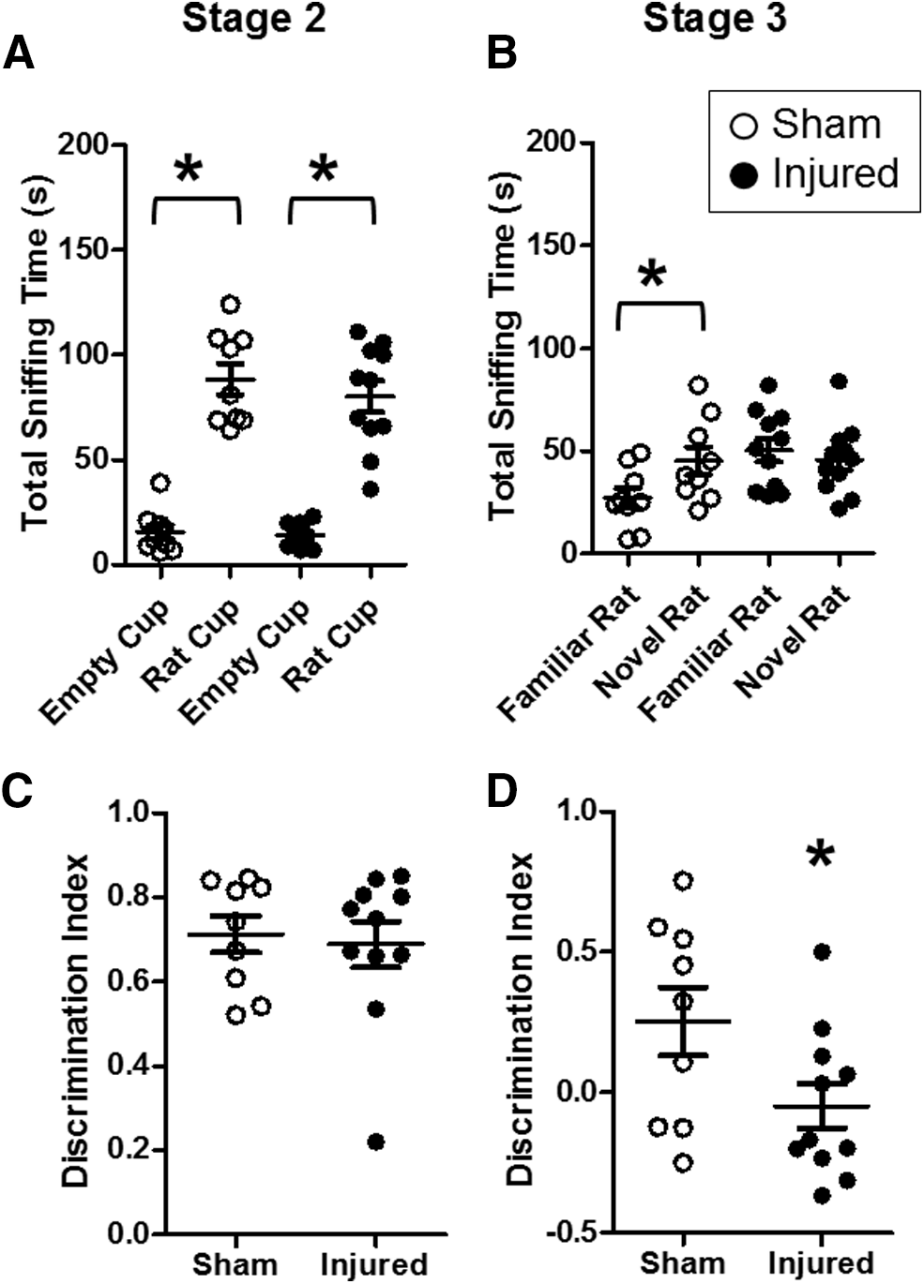
Pediatric TB1 impaired social recognition but not sociability during adulthood. Eleven-day-old male and female rat pups were tested for sociability (stage 2; ***A***, ***C***) and social recognition (stage 3; ***B***, ***D***) at eight weeks after injury (adult age) using the three-chamber test as described in Materials and Methods. Data are presented as time (in seconds) spent interacting with rat/object (***A***, ***B***) or as DI (***C***, ***D***). Open symbols represent sham animals, closed symbols represent injured animals. Bars represent group mean values, and the error bars represent SEM; *p < 0.05.

Brain injury on postnatal day 11 did not result in impairments in sociability (stage 2) in adulthood (Fig. 3*A*,*C*). Both sham-injured and brain-injured animals spent significantly more time interacting with the rat cup compared with an empty cup, as measured by total sniffing time (*F*_(1,32)_ = 140.5, *p* = 0.00; Fig. 3*A*). The discrimination ratio during stage 2 was not significantly different between sham-injured and brain-injured rats (*F*_(1,16)_ = 0.1, *p* = 0.7, ANOVA; Fig. 3*C*). The total time spent sniffing both stimuli during stage 2 also did not differ between sham and injured rats (data not shown; *F*_(1,16)_ = 0.5, *p* = 0.4, ANOVA). Brain-injured animals exhibited an impairment in social novelty recognition (stage 3) during adulthood (Fig. 3*B*,*D*). Statistical analysis revealed a significant interaction effect of injury status and the type of stimulus on total sniffing time (*F*_(1,32)_ = 4.1, *p* = 0.04; Fig. 3*B*). *Post hoc* tests showed that sham-injured animals spent significantly more time sniffing the novel rat compared with the familiar rat (*p* = 0.02), whereas brain-injured rats did not exhibit a significant difference in the time sniffing a novel rat compared with a familiar rat (*p* = 0.5). Statistical analysis also revealed that the DI during stage 3 was significantly lower in brain-injured animals compared with sham-injured animals (*F*_(1,16)_ = 4.6, *p* = 0.04, ANOVA; Fig. 3*D*). The total time spent sniffing both stimuli during stage 3 was slightly but significantly higher in brain-injured rats compared with sham-injured rats (data not shown; *F*_(1,16)_ = 5.6, *p* = 0.03, ANOVA). Overall, these experiments demonstrate that brain-injured male and female rats exhibit impairments in social recognition but intact sociability at eight weeks postinjury.

### Novel object recognition during adolescence and adulthood

**Figure 4.**
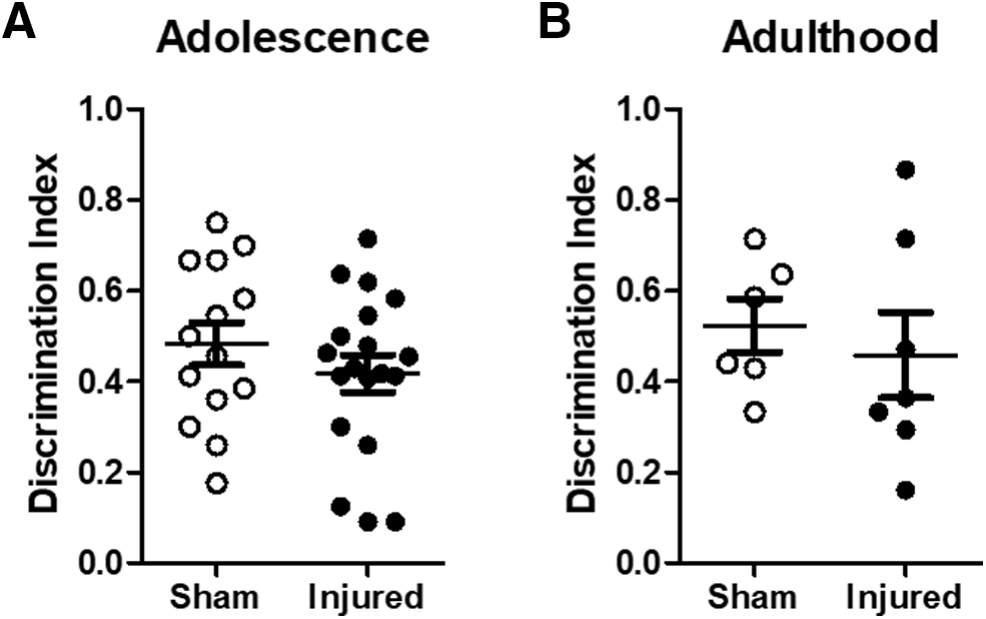
Brain-injured animals did not exhibit impairments in novel object recognition memory. At four weeks (adolescence, ***A***) and eight weeks (adulthood, ***B***) following injury, sham-injured and brain-injured animals were tested for novel object recognition memory as described in Materials and Methods. The DI was calculated as described in Materials and Methods. Open symbols represent sham animals, closed symbols represent injured animals. Bars represent group mean values, and the error bars represent SEM.

Brain injury did not result in an impairment in novel object recognition memory during either adolescence (Fig. 4*A*) or adulthood (Fig. 4*B*). Statistical analysis revealed no significant effect of injury status on the DI at either four weeks postinjury (*F*_(1,29)_ = 0.63, *p* = 0.43, ANOVA) or eight weeks postinjury (*F*_(1,9)_ = 0.22, *p* = 0.65, ANOVA). The sex of the animal did not have a significant effect on the DI at either four weeks postinjury (*F*_(1,29)_ = 0.19, *p* = 0.66, ANOVA) or eight weeks postinjury (*F*_(1,9)_ = 0.06, *p* = 0.81, ANOVA). Thus, these experiments confirm that TBI did not impair novel object recognition memory at either four or eight weeks postinjury.

### Expression of mRNA for OXT and OXTRs

**Figure 5.**
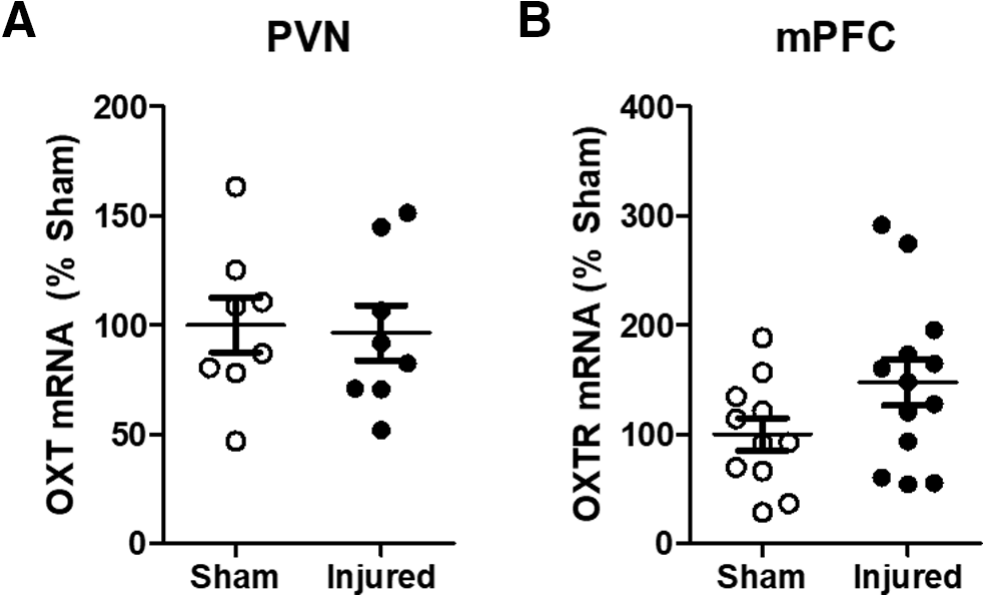
Expression of mRNA for OXT and OXTR in adolescence was not affected following TB1 in 11-d-old rats. After behavioral testing at four to five weeks postinjury, a subset of animals was euthanized and the expression of OXT (***A***) and OXTR (***B***) was evaluated in the PVN and mPFC, respectively. Open symbols represent sham animals, closed symbols represent injured animals. Expression (ddCT) values were normalized to sham values. Bars represent group mean values, and the error bars represent SEM.

To determine whether TBI induces changes in the OXT system, we measured OXT and OXTR mRNA within the PVN and PFC, respectively (Fig. 5*A*,*B*). Statistical analysis revealed no significant effects of injury (*F*_(1,19)_ = 0.25, *p* = 0.62, ANOVA) or sex (*F*_(1,19)_ = 0.04, *p* = 0.83) on OXT mRNA within the PVN (Fig. 5*A*). Similarly, there was no significant effect of injury (*F*_(1,20)_ = 2.11, *p* = 0.16, ANOVA) or sex (*F*_(1,20)_ = 0.22, *p* = 0.64, ANOVA) on OXTR mRNA within the PFC (Fig. 5*B*). Thus, TBI did not induce changes in OXT expression within the PVN or in OXTR expression within the PFC.

### Effects of OXT treatment on social behaviors and novel object recognition following TBI

**Figure 6.**
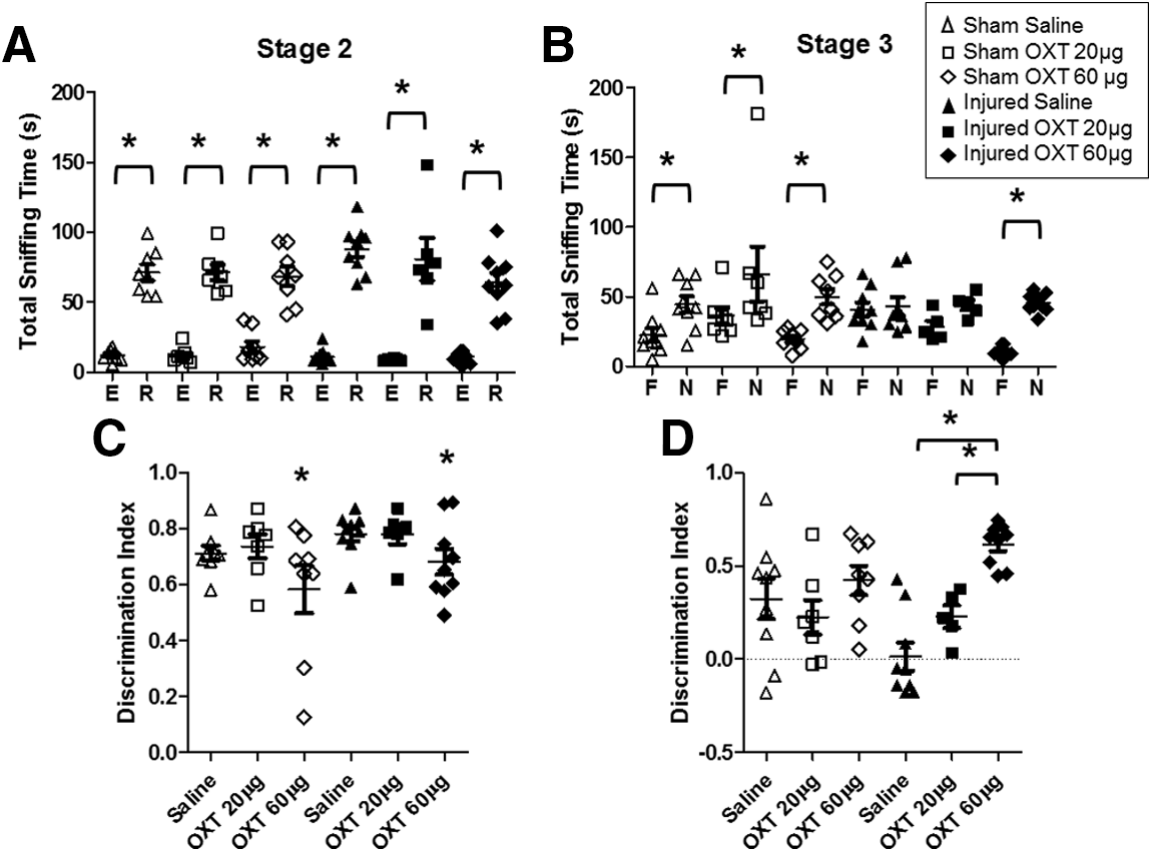
Intranasal administration of OXT administration reversed social recognition deficits in brain-injured animals in adolescence. At 1 hr before testing animals in the three-chamber test at four after injury, sham-injured and brain-injured animals were administered with saline, OXT at 20 μg, or oxy at 60 μg as described in Materials and Methods. ***A***, Seconds sniffing in stage 2. ***B***, Seconds sniffing in stage 3. ***C***, DI in stage 2. ***D***, DI in stage 3. E, empty cup; R, rat cup; F, familiar rat; N, novel rat. Open symbols represent sham rats, filled symbols represent injured rats, triangles represent vehicle-treated, squares represent 20-μg oxy-treated rats, diamonds represent 60-μg oxy-treated rats; *p < 0.05 in all panels. In panel ***C***, *p < 0.05 compared with brain-injured animals that received saline. Horizontal lines represent group mean values, and the error bars represent SEM.

To determine whether OXT treatment affects social behaviors after TBI, OXT (20 or 60 μg) was intranasally administered 1 hr before testing in the three-chamber test (Fig. 6). OXT treatment did not have a significant effect on sniffing times during stage 2 (sociability; Fig. 6*A*). Overall, rats spent more time sniffing the rat cup compared with the empty cup (*F*_(1,82)_ = 360.8, *p* = 0.00, ANOVA), regardless of injury status (*F*_(1,82)_ = 0.3, *p* = 0.5, ANOVA) or treatment (*F*_(2,82)_ = 0.9, *p* = 0.4, ANOVA). Interestingly, there was a significant effect of OXT treatment on the DI during stage 2 (*F*_(2,41)_ = 4.1, *p* = 0.02; Fig. 6*C*). *Post hoc* tests revelated that the DI was significantly lower in animals treated with 60 μg OXT compared with both saline (*p* = 0.02) and 20 μg OXT (*p* = 0.04), although these animals still spent more time sniffing the rat cup compared with the empty cup (Fig. 6*A*), indicating intact sociability. OXT treatment dose dependently increased social recognition (stage 3) in brain-injured animals (Fig. 6*B*,*D*). Statistical analysis of the DI scores revealed a significant interaction between injury status and treatment (*F*_(2,38)_ = 3.6, *p* = 0.03; Fig. 6*D*). *Post hoc* tests indicated that treatment with 60 μg OXT significantly increased the DI in brain-injured rats compared with brain-injured rats receiving either saline (*p* = 0.00) or 20 μg OXT (*p* = 0.02). OXT did not affect the total sniffing time during stage 3 (data not shown; *F*_(2,38)_ = 2.2, *p* = 0.1). OXT treatment did not affect novel object recognition memory (data not shown). Overall, these experiments demonstrate that OXT dose dependently increased social recognition in brain-injured animals without affecting non-social memory, with the higher 60 μg dose having the greatest effect on social recognition memory.

### Effects of OXT on novelty-induced locomotor activity

**Figure 7.**
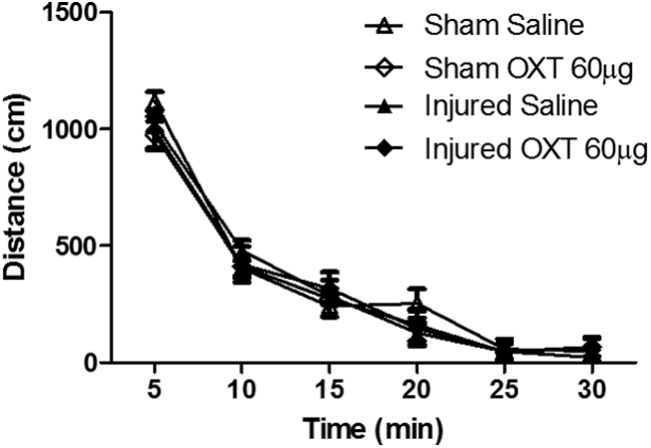
OXT administration did not affect novelty-induced locomotor activity. At five weeks after brain injury and following testing in the three-chamber test, sham-injured and brain-injured animals were tested for locomotor activity. Animals were administered either saline or OXT 60 μg as described in Materials and Methods.

An ANOVA with repeated measured revealed that there was no significant effect of brain injury on the distance traveled during a 30-min period (*F*_(1,30)_ = 0.09, *p* = 0.75; Fig. 7).

Treatment with 60 μg OXT 1 hr before locomotor testing also did not affect the distance traveled during the 30-min period (*F*_(1,30)_ = 0.4, *p* = 0.5, ANOVA). There was a main effect of time on the distance traveled (*F*_(5,150)_ = 257.7, *p* = 0.000, repeated measures ANOVA), with all animals exhibiting more activity during the first 5 min compared with the other time points (*p* < 0.001), confirming the assessment of novelty-induced activity. Thus, these experiments confirm that neither TBI nor OXT treatment affected novelty-induced locomotor activity.

### Whole-cell patch clamp electrophysiology

#### Intrinsic excitability of Layer II/III neurons

**Figure 8.**
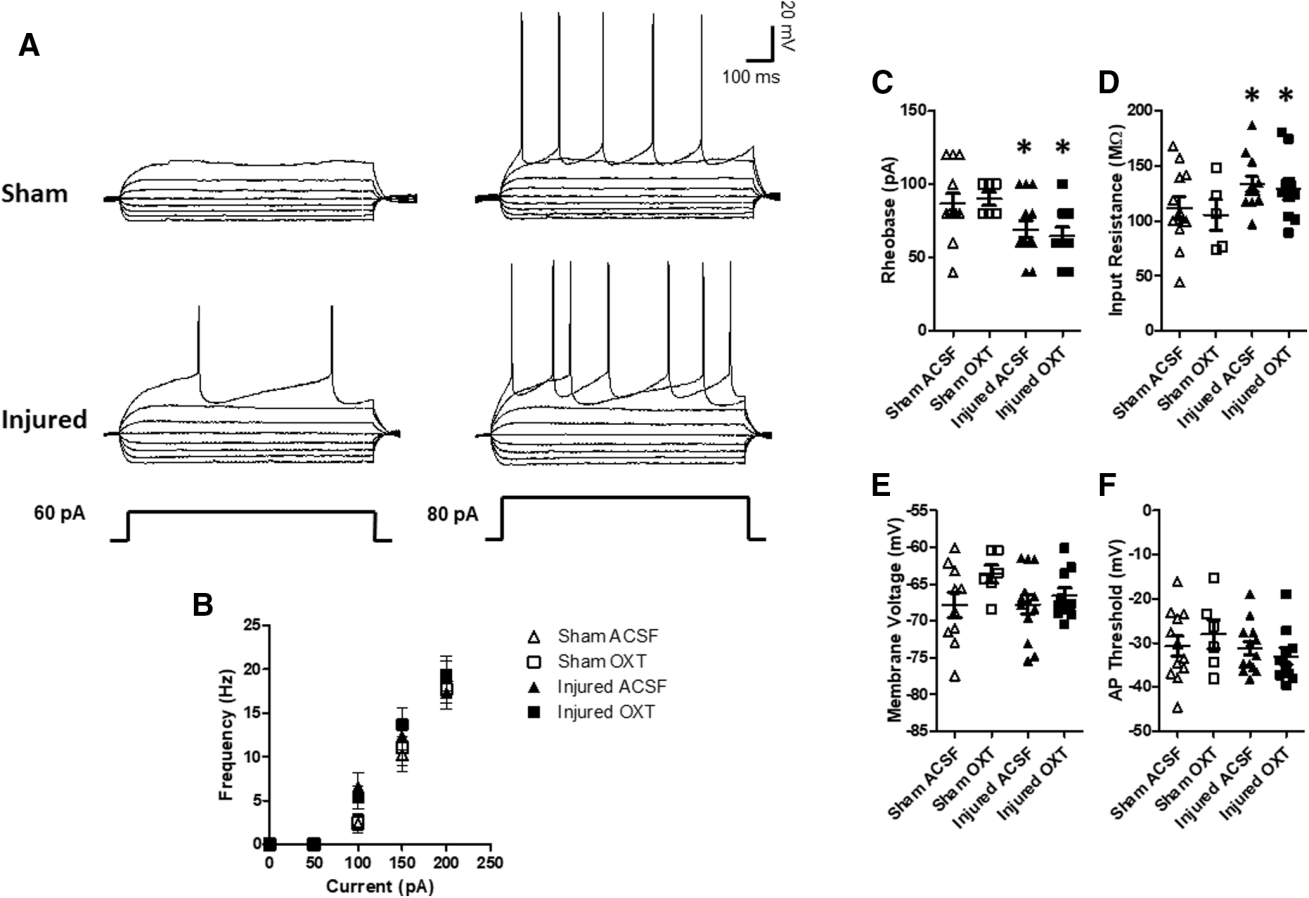
OXT did not affect membrane properties of Layer II/III pyramidal neurons within the mPFC. Following behavioral testing, slices containing the mPFC were obtained at six to seven weeks after injury and neuronal activity was measured using whole-cell patch clamp electrophysiology as described in Materials and Methods. ***A***, Representative current clamp traces from sham and brain-injured neurons. ***B***, Frequency of neuron firing in response to varying levels of current injection. ***C***, Rheobase. ***D***, Input resistance. ***E***, Membrane voltage. ***F***, Spike threshold. Bars represent mean group values, and error bars represent SEM. Open triangles represent sham cells bathed with aCSF (*N* = 12), open squares represent sham cells bathed with 1 μm OXT (*N* = 6), filled triangles represent injured cells bathed with aCSF (*N* = 13), and filled squares represent injured cells bathed with OXT (*N* = 10); *p < 0.05.

Current clamp recordings from pyramidal cells within the mPFC from sham and brain-injured animals (illustrated in Fig. 8*A*) reveled a significant effect of TBI on input resistance (*F*_(1,35)_ = 4.6, *p* = 0.03, ANOVA; Fig. 8*D*). The input resistance was significantly higher in neurons from brain-injured animals compared to their sham-injured counterparts (*p* = 0.03) and was not affected by OXT (*F*_(1,35)_ = 0.3, *p* = 0.6, ANOVA). An increase in input resistance is typically associated with an increase in neuronal excitability. There was also a significant effect of TBI on the rheobase (*F*_(1,36)_ = 9.7, *p* = 0.003, ANOVA; Fig. 8*A*,*C*), which was significantly decreased in neurons from brain-injured animals compared to sham animals (*p* = 0.003), indicative of increased excitability, and was similarly not affected by OXT (*F*_(1,36)_ = 0.01, *p* = 0.9, ANOVA). However, there was no difference in the spike frequency in response to varying levels of current injection between the groups (*F*_(2,27)_ = 0.7, *p* = 0.53, repeated measures ANOVA; Fig. 8*B*). There were no differences between cells recorded from sham and brain-injured animals in the membrane voltage (*F*_(1,35)_ = 0.97, *p* = 0.3, ANOVA; Fig. 8*E*), spike threshold (*F*_(1,37)_ = 0.1.5, *p* = 0.2, ANOVA; Fig. 8*F*), or spike amplitude (*F*_(1,37)_ = 3.0, *p* = 0.09, ANOVA; data not shown). Overall, these experiments indicate that TBI resulted in an increase in the excitability of Layer II/III pyramidal cells within the mPFC which was not affected by OXT.

#### Excitatory inputs to Layer II/III neurons

**Figure 9.**
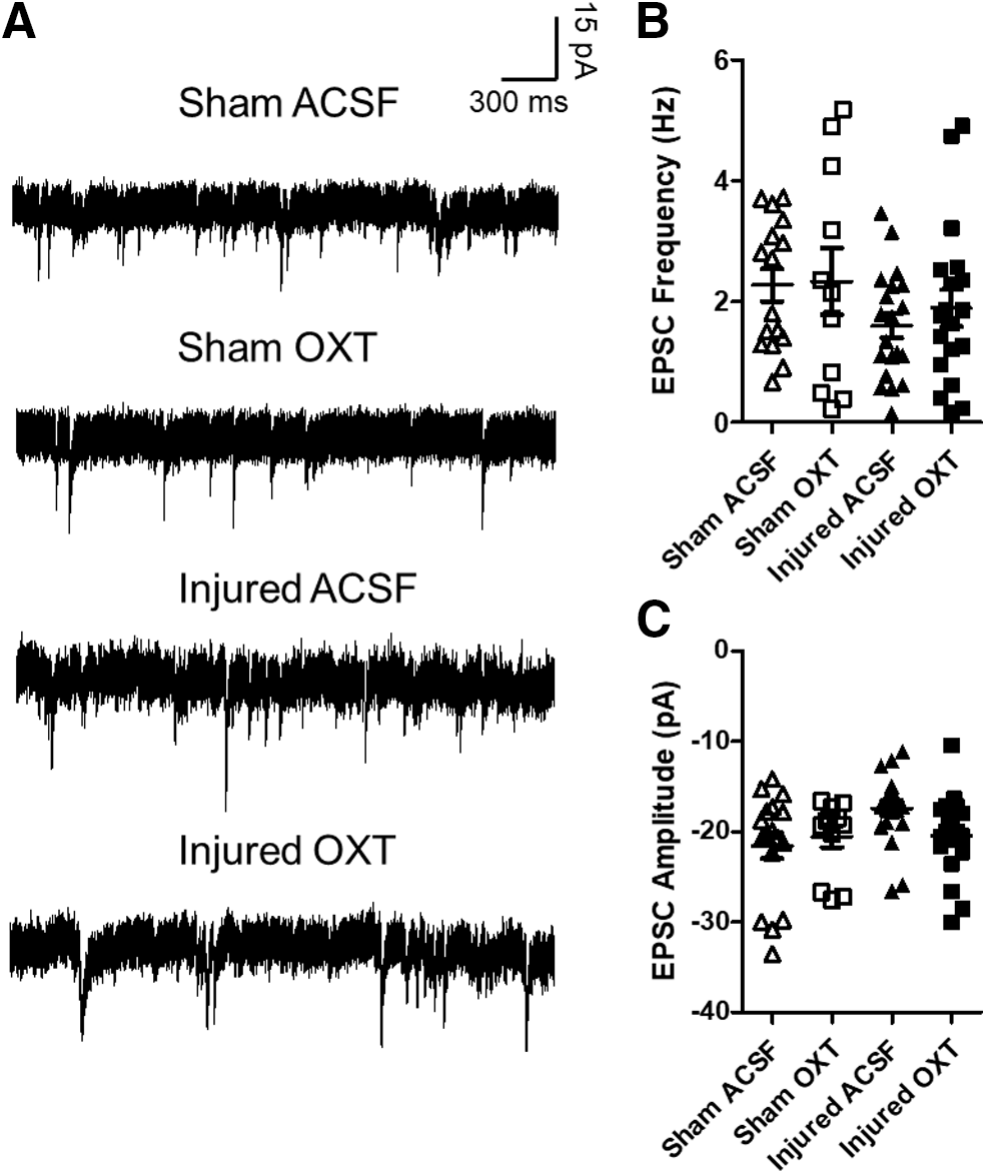
OXT did not affect spontaneous EPSCs in Layer II/III neurons within the mPFC. ***A***, Representative traces of spontaneous EPSCs recorded from Layer II/III pyramidal neurons within the mPFC. ***B***, Frequency of spontaneous EPSCs. ***C***, Amplitude of spontaneous EPSCs. Bars represent group mean value, and error bars represent SEM. Open triangles represent sham cells bathed with aCSF (*N* = 16), open squares represent sham cells bathed with 1 μm OXT (*N* = 11), filled triangles represent injured cells bathed with aCSF (*N* = 20), filled squares represent injured cells bathed with 1 μm OXT (*N* = 19).

Representative traces of spontaneous EPSCs are illustrated in Figure 9*A*. There were no significant differences in either the frequency (*F*_(1,58)_ = 3.1, *p* = 0.08, ANOVA; Fig. 9*B*) or amplitude (*F*_(1,60)_ = 2.3, *p* = 0.1, ANOVA; Fig. 9*C*) of spontaneous EPSCs between sham-injured cells and brain-injured cells recorded with either aCSF or OXT. Thus, there were no significant effects of either TBI or OXT application on excitatory inputs to Layer II/III neurons.

#### Inhibitory inputs to Layer II/III neurons

**Figure 10.**
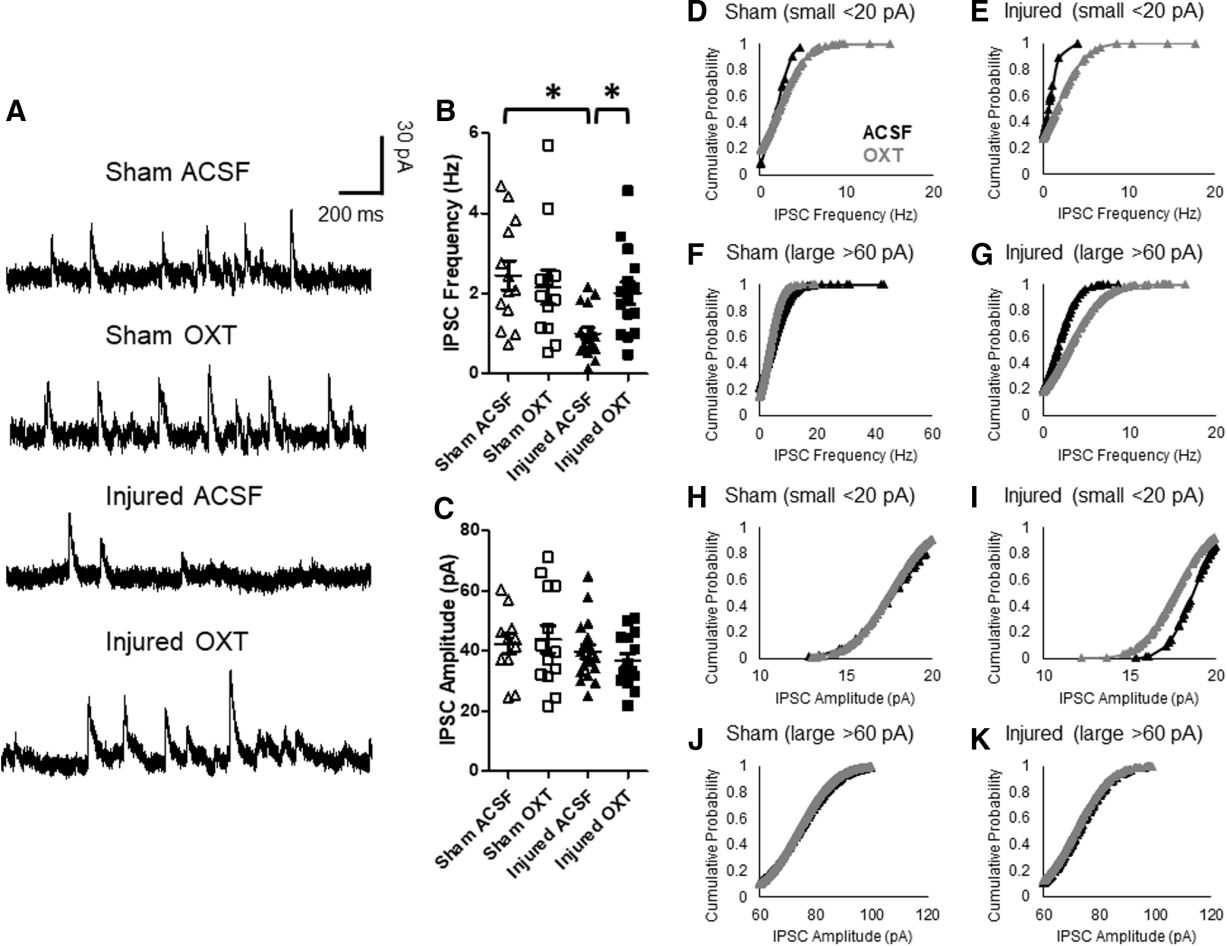
OXT increased the frequency of spontaneous IPSCs in Layer II/III neurons within the mPFC. ***A***, Representative traces of spontaneous IPSCs recorded from Layer II/III pyramidal neurons within the mPFC. ***B***, Frequency of spontaneous IPSCs. ***C***, Amplitude of spontaneous IPSCs. Bars represent group mean value, and error bars represent SEM. Cumulative probability of small IPSC frequencies in sham (***D***) and injured cells (***E***). ***F***, ***G***, Cumulative probability of large IPSC frequencies in sham (***F***) and injured cells (***G***). Cumulative probability of small IPSC amplitudes in sham (***H***) and injured cells (***I***). Cumulative probability of large IPSC amplitudes in sham (***J***) and injured cells (***K***; Ryan et al., 2016; Douglas, 2020). Open triangles represent sham cells bathed with aCSF (*N* = 13), open squares represent sham cells bathed with 1 μm OXT (*N* = 12), filled triangles represent injured cells bathed with aCSF (*N* = 17), filled squares represent injured cells bathed with 1 μm OXT (*N* = 15); *p < 0.05.

Representative traces of spontaneous IPSCs are illustrated in Figure 10*A*. There was a significant interaction effect on the frequency of spontaneous IPSCs (status × treatment: *F*_(1,49)_ = 5.6, *p* = 0.02, ANOVA). *Post hoc* tests revealed that IPSC frequency was significantly lower in cells from brain-injured animals recorded with ACSF relative to cells from sham-injured animals (*p* = 0.008) and cells from brain-injured animals recorded in the presence of OXT (*p* = 0.02; Fig. 10*B*). There was no difference in the IPSC frequency in sham cells bathed with either aCSF or OXT (*p* = 0.49). However, there were no effects of TBI (*F*_(1,49)_ = 1.3, *p* = 0.25, ANOVA) or OXT (*F*_(1,49)_ = 0.0006, *p* = 0.98, ANOVA) on the amplitude of spontaneous IPSCs (Fig. 10*C*). There were no effects of sex on the frequency (*F*_(1,49)_ = 0.64, *p* = 0.4, ANOVA) or amplitude (*F*_(1,49)_ = 2.7, *p* = 0.1, ANOVA) of IPSCs recorded from Layer II/III.

Because IPSCs evoked by SOM-expressing interneurons, which typically project to distal dendrites of pyramidal neurons (Urban-Ciecko and Barth, 2016), are smaller compared with parvalbumin-expressing interneurons (Holley et al., 2019), cumulative probability analyses on the amplitudes and frequencies of either small (<20 pA) or large (<60 pA) IPSCs were performed. A Kolmogorov–Smirnov test revealed a significant difference in the distribution of frequencies of both small IPSCs (D_(43)_ = 0.67, *p* < 0.001) and large IPSCs (D_(572)_ = 0.33, *p* < 0.001) between sham and injured mPFC cells bathed with aCSF (Fig. 10*D–G*). Bath application of OXT led to a rightward shift in the distribution of frequencies of both small IPSCs (D_(140)_ = 0.37, *p* < 0.005) and large IPSCs (D_(428)_ = 0.27, *p* < 0.001) in brain-injured cells (Fig. 10*E*,*G*) but did not affect either small IPSCs (D_(204)_ = 0.33, *p* > 0.1) or large IPSCs (D_(744)_ = 0.06, *p* > 0.1) in sham cells (Fig. 10*D*,*F*). There was no effect of TBI on the amplitude of either small IPSCs (D_(75)_ = 0.27, *p* > 0.1) or large IPSCs (D_(349)_ = 0.08, *p* > 0.1) in cells bathed with aCSF, although bath application of OXT led to a leftward shift in the distribution of amplitudes of small IPSCs in brain-injured cells (D_(205)_ = 0.36, *p* < 0.001; Fig. 10*I*), without affecting large IPSC amplitudes (D_(436)_ = 0.13, *p* > 0.1; Fig. 10*K*). Moreover, OXT did not affect the amplitudes of either small IPSCs (D_(236)_ = 0.22, *p* > 0.1) or large IPSCs (D_(1150)_ = 0.05, *p* > 0.1) in sham cells (Fig. 10*H*,*J*).

Overall, these experiments indicate that TBI resulted in a significant decrease in the frequency of IPSCs recorded from Layer II/III pyramidal neurons within the mPFC, and that bath-application of OXT increased the IPSC frequency in pyramidal cells from brain-injured animals without having an overall effect on IPSC amplitude. Moreover, OXT increased the frequency of IPSCs of both small and large amplitudes but had a selective effect on the amplitudes of small IPSCs in mPFC neurons from brain-injured animals.

## Discussion

The present study demonstrates that moderate TBI in the 11-d-old rat resulted in social recognition deficits at four and eight weeks postinjury, corresponding to adolescence and adulthood, respectively. Intranasal administration of OXT before behavioral testing reversed social recognition deficits in brain-injured animals. Closed-head injury did not affect sociability in the three-chamber test or novel object recognition memory, indicating that these deficits were specific to the neural circuits underlying social memory rather than recognition memory in general. Moreover, brain-injured animals exhibited a significant increase in excitability and decrease in the frequency of spontaneous IPSCs in Layer II/III PFC pyramidal neurons within the mPFC, which was consistent with previous results (Lengel et al., 2020) Bath application of OXT increased IPSC frequency but did not affect neuronal excitability. Overall, these findings suggest that OXT improves social recognition memory following pediatric TBI, and that this effect may be mediated by the facilitation of inhibitory neurotransmission within the PFC by OXT.

The findings from this study differ from previous studies demonstrating changes in social behaviors following pediatric TBI. Semple et al. (2012) found that contusive brain trauma in 21-d-old mice resulted in deficits in sociability and preference for social novelty during adulthood (p60-p70), but not adolescence (p35–p42). Another study reported deficits in both sociability and social novelty preference during adolescence (p31) following CCI in 14-d-old rats (Wei et al., 2016). These seemingly inconsistent results are likely because of a variety of factors including age at time of injury, injury model, and species. While we did not observe sex differences in the effects of TBI on social behaviors, Semple et al. (2017) found that while male mice injured on postnatal day 21 exhibited deficits in both sociability and preference for social novelty in adulthood, female mice exhibited reduced sociability but intact preference for social novelty. Moreover, these sex-dependent changes were associated with a reduction in dendritic complexity within the PFC and hippocampus that was more apparent in males and preceded the onset of social impairments (Semple et al., 2017). Although evidence of neuronal death or neurodegeneration within the mPFC, has not been observed (Lengel et al., 2020), changes in dendritic morphology following TBI may be a potential mechanism underlying changes in the excitatory/inhibitory balance within the mPFC.

Our results demonstrate that OXT improves social recognition following TBI, which corroborate previous evidence that improvements in social behaviors mediated by stem cell transplantation following CCI are associated with higher levels of OXT and OXTR (Wei et al., 2016). The lack of an effect of OXT on social behaviors in sham animals in the current study supports previous findings in which OXT did not affect social novelty in wild-type mice and only increased preference for social novelty in mice that exhibit autism-like behaviors (Zhang et al., 2016; Hara et al., 2017). The ability of OXT to improve social recognition in brain-injured animals may be related to its ability to regulate GABAergic activity. TBI resulted in a reduction in the frequency but not amplitude of spontaneous IPSCs in the mPFC, which could implicate presynaptic changes in inhibitory neurotransmission. An imbalance of the cortical excitation/inhibition balance has been previously implicated in adult TBI (Witgen et al., 2005; Bonislawski et al., 2007; Ding et al., 2011; Cantu et al., 2015; Carron et al., 2016; Brizuela et al., 2017; Witkowski et al., 2019) and has been linked to reductions in inhibitory neurotransmission (Witgen et al., 2005; Bonislawski et al., 2007; Cantu et al., 2015; Carron et al., 2016). OXTRs within the PFC are expressed on regular-spiking SOM neurons (Nakajima et al., 2014), which synapse with pyramidal neurons in Layer II/III (Urban-Ciecko and Barth, 2016). Although reductions in the numbers of SOM-containing inhibitory interneurons have been reported following moderate TBI in adult rats (Carron et al., 2020), a previous study reported that pediatric TBI does not result in neuron loss in the mPFC (Lengel et al., 2020).

Bath application of OXT in the present study was found to increase the IPSC frequency but not amplitude in brain-injured PFC slices, suggesting that the effect of OXT on IPSCs were likely mediated through the regulation of GABA release from inhibitory interneurons synapsing with pyramidal neurons, rather than acting directly on OXTRs on pyramidal neurons. These effects of OXT were specifically observed in pyramidal neurons from brain-injured but not sham slices. Although the effects of OXT on IPSC frequency in PFC pyramidal neurons have not previously been investigated, a previous study reported an increase in IPSC frequency recorded from mossy cells in the dentate gyrus with application of the OXTR agonist, TGOT (Harden and Frazier, 2016). In part, this differential response to TGOT may reflect its higher specificity for the OXTR over the vasopressin (AVP) receptor compared with OXT (Harden and Frazier, 2016). This observation also suggests that OXTRs in the mPFC may be exhibit higher sensitivity for OXT following TBI, possibly because of a decrease in basal OXT levels, resulting in greater OXTR activation by exogenous OXT in injured cells relative to sham cells.

Because OXTRs are predominantly expressed on SOM neurons in the mPFC (Nakajima et al., 2014), which evoke smaller IPSCs in pyramidal neurons compared with parvalbumin neurons (Holley et al., 2019), we predicted that OXT would preferentially affect the frequency and/or amplitude of small IPSCs. While OXT significantly increased the frequency of both small and large IPSCs in injured cells, it selectively affected the amplitudes of small IPSCs resulting in a leftward shift in the distribution of small IPSC amplitudes recorded from injured cells, suggestive of an increase in the number of smaller currents relative to larger currents. The beneficial effects of OXT on social recognition may therefore be a result of increasing GABAergic release from terminals of GABAergic neurons, including SOM and likely parvalbumin interneurons as well. Thus, although beyond the scope of this study, measuring the direct effects of OXT bath application on the activity of SOM and parvalbumin interneurons following TBI will be an important area of future investigation.

In contrast to a previous study (Lengel et al., 2020), we did not observe changes in spontaneous EPSC frequency in Layer II/III pyramidal neurons in the mPFC. This may be because of differences in the timing of electrophysiological recordings, which were conducted at the conclusion of behavioral testing (between six and seven weeks postinjury) in the current study. However, we did observe an increase in neuronal excitability (increase in input resistance and decrease in rheobase) and a reduction in spontaneous IPSC frequency, corroborating our previous findings and suggesting the effects of TBI on Layer II/III EPSCs may be transient. In contrast, our current data indicate that the effects of TBI on Layer II/III IPSCs are sustained up to seven weeks following injury. We previously identified changes in the excitatory amino acid transporter 3 (EAAT3) and voltage-gated sodium channel β3-subunit (NaVβ3) as potential mechanisms underlying the effects on cellular function by progesterone treatment during the first week following injury (Lengel et al., 2020). In the present study, because the OXT was administered 1 hr before behavioral testing, it is more likely that its effects were mediated through acute changes in signal transduction, rather than transcriptional changes or anti-inflammatory effects of OXT. Furthermore, whereas progesterone was found to predominantly affect neuronal excitability and excitatory neurotransmission, the findings from this study suggest that the behavioral effects of OXT may be specifically mediated through its effects on inhibitory neurotransmission (Harden and Frazier, 2016).

We did not observe changes in OXT mRNA in the PVN or OXTR mRNA in the PFC. Although expression of OXT and OXTR proteins were not measured in the current study, a previous study reported no changes in OXT or OXTR protein concentrations at two weeks following TBI in 14-d-old rats (Wei et al., 2016). Nonetheless, TBI may influence important developmental processes that occur during the first postnatal weeks. Notably, the maturation of OXT-producing neurons in the rat hypothalamus occurs later compared with other neuropeptides including AVP and SOM, with OXT mRNA levels reaching about half of the adult levels during the third postnatal week (Almazan et al., 1989). Moreover, this upregulation in gene expression occurs concomitantly with the establishment of synaptic input to the PVN and the development of output projections from the PVN to target regions during the first two postnatal weeks (Almazan et al., 1989). Thus, it is possible that TBI leads to a loss of PVN projections to the mPFC, resulting in a reduction in OXT release in the mPFC and, in turn, a decrease in the basal activation of OXTRs on these neurons. A loss of basal OXT transmission in the mPFC could also explain the selective effect of OXT administration in injured but not in sham cells, where there may be higher levels of endogenous OXT release.

Although changes in OXT or OXTR mRNA were not observed at six weeks after injury, we cannot rule out the possibility of transient changes in TBI-induced expression of these genes at earlier time-points following injury. The hallmark of pediatric TBI is diffuse (traumatic) axonal injury (Kannan et al., 2014) which has been validated in our model (Raghupathi and Huh, 2007). Moreover, although histopathological damage within the mPFC following pediatric TBI has not been observed (Lengel et al., 2020), it is possible that the PVN OXT neurons that project to the mPFC may be injured, resulting in impaired transmission of OXT to the mPFC and/or other areas involved in social recognition. It is also important to acknowledge the existence of cross-reactivity between OXT and other neuropeptide systems such as AVP (Song and Albers, 2018), and thus the possibility that the beneficial effects of OXT treatment may have been partly mediated by AVP receptors. Thus, the molecular mechanism underlying the effects of OXT treatment on social recognition deficits following closed head injury remain a topic for further exploration.

In the present study, we did not observe an impairment in novel object recognition memory following a single closed-head injury in the 11-d-old rat, which differs from previously published data in the neonate rat (Lengel et al., 2020). This discrepancy is likely because of differences in the testing conditions; in the present study animals were tested for novel object recognition under dark conditions, in contrast to the previous study in which they were tested under normal light conditions. Dark lighting conditions were used for novel object recognition testing in the present study to maintain the consistency of testing conditions between the novel object recognition test and three-chamber test. We chose to test animals in the three-chamber test in dark conditions to avoid anxiogenic effects of excessive room lighting, which can influence the activity of animals behaving in the three-chamber test (Kaidanovich-Beilin et al., 2011).

A limitation of this study is that we did not measure changes in olfaction following TBI. Thus, we cannot rule out the possibility that the observed deficits in social novelty recognition may have been influenced by impairments in olfactory discrimination in brain-injured animals. However, the absence of deficits in sociability (stage 2) would suggest that olfaction may not be adversely affected by TBI. Moreover, a similar study reported sensory deprivation-induced social memory deficits despite normal olfaction, sociability, locomotor activity, and novel object recognition memory (Zhang et al., 2016), confirming that impairments in social behaviors can occur independent of impairments in olfaction.

Overall, these findings demonstrate that a closed head injury in 11-d-old rats results in social recognition deficits which are accompanied by alterations in neuronal functionality within the mPFC. Further, we have identified the regulation of GABAergic neurotransmission within the PFC as a potential mechanism of these effects of OXT on social behaviors. Intranasal OXT treatment has shown promise in improving social deficits in children with autism (Parker et al., 2017). To the best of our knowledge, this is the first study to demonstrate beneficial effects of OXT administration on social behavioral outcomes following TBI in pediatric animals. Our findings provide support for the potential of intranasal OXT treatment as an effective therapeutic strategy for social deficits following pediatric TBI.

## Synthesis

Reviewing Editor: Viji Santhakumar, University of California, Riverside Decisions are customarily a result of the Reviewing Editor and the peer reviewers coming together and discussing their recommendations until a consensus is reached. When revisions are invited, a fact-based synthesis statement explaining their decision and outlining what is needed to prepare a revision will be listed below. The following reviewer(s) agreed to reveal their identity: Akiva Cohen.

The study examines changes in social novelty recognition after pediatric experimental brain injury to understand potential mechanisms leading to increased risk for social behavioral deficits in children after brain injury. The studies use a combination of well-justified behavioral tests to identify specific deficits in social novelty detection after “severe” brain trauma in young rats using the cortical impactor. This specific behavioral deficit is seen at all time points studied and is strikingly corrected with intranasal oxytocin therapy, a hormonal neuropeptide well-known to modulate social behaviors. Since the PFC is implicated in social behaviors, the authors examine PFC neuron excitability and spontaneous excitatory and inhibitory synaptic inputs and identify changes in rheobase and reduced IPSCs. While oxytocin perfusion did not alter intrinsic physiology, it partially reversed changes in IPSC frequency. The data are interesting and support the conclusions. However, some aspects of the manuscript including experimental details of the model implementation, physiological experiments and analysis need to be improved. Additionally, the figures need to be substantially improved in quality and representative recordings included for physiology studies. I have included below the major issues that would need to be addressed in a revision. I am also including detailed reviewer comments below the synthesis.

1. Additional behavioral data to compare social recognition testing (and OXT sensitivity) in animals injured at P11 vs. 4-6 weeks (young adulthood) would be important to establish whether any of these changes are unique to “pediatric TBI.” While the whole battery or tests are not required, the social recognition test showing significant changes in this group would be helpful. As it stands, the data do not provide any evidence that these results are dependent on age at time of injury, an emphasis point in their paper.

2. The details on the description of the electrophysiological recordings and analysis is lacking. Specifically, how intrinsic parameters were measured and analyzed needs to be detailed. Was the input resistance measured from the slope fit to subthreshold input-output plots? What was the duration of the current step used to determine rheobase? How was AP threshold determined? The details in the methods need to be expanded. Since cells were not labeled for morphology, the selection criteria used to ensure that interneurons were not included in the data need to be stated.

3. Representative traces of I/V relationships and input-output plots illustrating firing frequency at various current steps need to be included.

4. It is important to examine oxytocin effects on IPSCs in control/sham cells. This will enable evaluation of whether the oxytocin effects are specific to injury or would be present otherwise. This will also enable appropriate two way ANOVA testing for effect of injury drug and interactions. This is especially important because OXT had no effects on behavior in sham/control animals. It would also add general information on the characteristics and magnitude of this effect (e.g. is OXT a convenient and reliable way to bolster IPSC frequency in cortical neurons?). Clarification of the timing, duration of OXT exposure in the physiology experiments is needed; were the effects of oxytocin acute or were slices treated with oxytocin for longer periods?

5. The methods for EPSC IPSC recordings as currently detailed in the methods are incorrect. EPSCs and IPSCs cannot be isolated using K-gluconate based internals without blockers and cells cannot be held at 0 mV in K-Gluc. The appropriate methods need to be provided to ensure that EPSCs and IPSCs were correctly isolated. In this regard, bath application of oxytocin improves rather than reverses changes in IPSC frequency, and overstating this finding as reversal should be avoided.

6. The discrepancy between timepoint for behavioral and physiology studies needs to be discussed.

7. Discussion/Interpretation of physiology data needs to be enhanced.

8. The experimental model needs to be better defined in terms of injury severity. The description of ‘closed head injury’; while strictly true, lacks specificity. Inclusion of additional descriptions of the injury and the location of the injury with respect to PFC involvement would be important. Potential impact on olfaction needs to be controlled for, especially in the context of social behaviors.

9. The figure quality is unacceptable in its current form and needs to be improved. Addition of schematic of behavioral set ups to the relevant figures and representative traces to physiology data is needed. Scales on the discrimination index need to be kept constant across figures to allow better comparisons.

Reviewer 1:

"Intranasal administration of oxytocin attenuates social recognition deficits and increase prefrontal cortex inhibitory postsynaptic currents following traumatic brain injury"

This manuscript describes results using a model of severe traumatic brain injury (TBI) in young, “pediatric” rats (skull fracture, cerebral hematoma). The authors’ focus is largely on behavior alterations after this pediatric injury and data is presented to indicate specific deficits in social recognition after this early life TBI (i.e. discrimination of novel vs. familiar rat), without any apparent changes in episodic memory (novel object recognition), social behavior (preference for rat over inanimate object), or locomotor ability. This specific behavioral deficit is seen at all time points studied and is strikingly corrected with intranasal oxytocin therapy, a hormonal neuropeptide well-known to modulate social behaviors. The authors performed cellular physiology on layer 2/3 pyramidal neurons in prefrontal cortex (PFC), a cortical area believed to influence social behaviors, and report changes in excitability (rheobase) after TBI without changes in glutamatergic synaptic inputs or passive membrane properties, and a reduction in GABAergic synaptic input that is corrected with oxytocin therapy.

Manuscript is well-written, the methods are appropriate, data appear of high quality and generally support their conclusions. Overall, additional data and analyses and expanded discussion would significantly strengthen the manuscript. I have several specific comments listed below.

Major points:

1. The authors throughout refer to ‘closed head injury’; while strictly true, this is a somewhat generic term that can also applied to mild TBI/concussion. It may be preferable to refer to the injury as severe TBI based on the descriptions of the injury.

2. Inclusion of additional descriptions of the injury would be helpful, I presume based on descriptions that the PFC is not directly involved (?).

3. The authors provide nice discussions and emphasize the variable behavioral changes observed after TBI, variabilities that authors speculate may relate to age at time of injury and motivating the study of rats injured at a young age. Direct comparison in this specific model of social recognition performance in animals injured at older ages to those at injured at P11 (and assessment of oxytocin responsiveness) would be real progress on age-dependent outcomes after TBI.

4. Figure 7. Including illustrations of representative recordings from sham, TBI, and TBI+OXT cells would improve this figure, specifically to highlight the increased excitability of neurons from injured animals (lower rheobase value) that is independent of changes in passive membrane properties. Assessment of rheobase often involves input-output curves for current injection and spike #, this approach also allows interpretation of changes in firing pattern/peak firing rates. Were there changes in peak firing rates of firing patterns in layer 2/3 PFC cortical neurons after injury?

5. Although the major focus of paper is on behavioral changes, very little attention is given to interpretation and discussion to their cellular physiology data. How do the authors imagine the change in rheobase occurs in the absence of changes in input-resistance, AP threshold? Their data on IPSCs could be consistent with both pre- and post-synaptic effects of TBI but OXT only corrected IPSC frequency. Is that correct? Do the authors conclude that there is dysfunction/loss of SS interneurons in PFC after TBI and OXT somehow corrects this? Expanded discussion on these points will be helpful.

6. Clarification of the timing, duration of OXT exposure in the physiology experiments is needed; were the effects of oxytocin acute or were slices treated with oxytocin for longer periods? Additionally, determining the effects of oxytocin in sham/control cells seems important. Is this effect unique to injured rats? One might hypothesize that the OXT effect on IPSCs would be even larger in sham/control animals when there is no interneuronal dysfunction.

7. Although it may be beyond scope of this manuscript, recording from interneurons and assessment of OXT effects in sham/TBI animals would be a significant advancement. Have the authors attempted to record from interneurons? Based on literature is there an established effect of OXT on interneurons or a response to OXT that could be assessed with cellular physiology? The authors do note that SST interneurons have increased excitability and increased glutamatergic transmission; are these properties that are defective after TBI and corrected by OXT?

8. Inclusion of additional descriptions of OXT action, specific signal transduction systems activated, and characteristics of OXT release would be helpful to this reader. Although TBI did not alter OXT or OXT-R mRNA levels in PFC, are there any literature or observations of the authors to suggest that regulated release of OXT is altered after TBI? Diurnal v. nocturnal differences that could relate to the cited differences in social behavior when tested in light/dark? Does social behavior change in sham/control depending on whether testing done in light/dark environment?

9. Discussion. Authors reference potential anti-inflammatory effects of oxytocin. Do the authors believe these anti-inflammatory effects of oxytocin (and the time course of these effects) played a role in their results given the timing of oxytocin administration?

Minor points:

1. Abstract. Including additional descriptions or reference to the severity and type of injury in the literature review of TBI/social deficits in the abstract would be helpful. Is there a “dose-dependence” to this effect or does mild TBI in pediatric pts/young animals produce similar deficits as severe TBI?

2. Figures. The resolution of illustrations could be improved, the versions I have for review are highly-pixellated. This may be corrected when figures are appropriately sized but an item for the authors to address. Including symbol legends in the figures themselves could also be considered to aid interpretation, this is particularly true for Fig. 5A, B (symbols are explained in legends but in-figure legends may be helpful).

3. Results, “effects of TBI...during adulthood.” Consider changing first sentence in this section to clarify that these “pediatric"-injured rats studied at later ages; as is this sentence could be interpreted to indicate injury occurred in adult animals.

4. Is table 1 necessary?

5. Inclusion of page numbers and line numbers on manuscript will be helpful.

Reviewer 2:

After traumatic brain injury (TBI), young children are more likely to show deficits in social skills later in life. The hormone oxytocin is highly associated with social behaviors and might be used as a treatment to improve social behavior deficits after TBI. To test this hypothesis, the authors evaluated social behaviors in rats 4 and 8 weeks after experimental TBI performed at postnatal day (PND) 11, and some rats received intranasal oxytocin as a treatment. The authors found that injured rats showed dose-dependent improvements in social behavior tests with the administration of oxytocin. The authors then performed electrophysiology experiments in the medial prefrontal cortex (mPFC), where increases in excitability have previously been shown to result in less social behavior. The authors found that after injury, layer 2/3 pyramidal cells in the mPFC showed a decreased rheobase and a decreased IPSC frequency and amplitude, indicating that these cells were more excitable after injury. With the bath application of oxytocin, IPSC frequency increased, providing some evidence that reducing excitability may be part of the mechanism of oxytocin in the brain. The authors also tested for changes in mRNA expression of oxytocin peptide in the paraventricular nucleus and oxytocin receptors in the mPFC after injury. They observed no changes, indicating that any observed effects of TBI related to oxytocin were not due to changes in peptide or receptor expression levels in those brain regions.

The reviewer would like to commend the authors for presenting a paper that is easy to read, with a clear storyline and interesting conclusions supported by different methods of data collection.

Major Comments:

• In the discussion, the authors state that the oxytocin administration ‘reverses’ certain alterations associated with TBI. While this is somewhat true of the stage 3 behavior, it is not true of the IPSC frequency. The bath application of oxytocin may improve IPSC frequency, but it does not ‘reverse’ the effects of TBI. This language overstates the conclusions and should be changed.

• The authors do not describe the injury severity in their paper (e.g., mild, moderate, severe).

• The authors should explain their use of cyclophilin-A as a reference gene for the qPCR experiments. Since protein expression may be altered after TBI, the authors should at a minimum provide justification for the choice of cyclophilin-A over other common reference genes (e.g., GAPDH, beta-actin), and at a minimum offer evidence that it is a stably expressed protein after TBI and therefore an appropriate reference.

• The authors should describe the methods used to calculate the electrophysiological measurements of input resistance, resting membrane potential, rheobase, action potential threshold, and action potential amplitude, rather than stating that they were ‘analyzed’.

• There is a possibility that head injury may have altered olfaction rather than sociability, thereby altering the behavioral findings. The authors should offer a control to show that this is not the case.

• The authors state that the animals were habituated to the testing environments for the behavioral tests, but not whether they were handled by the experimenters prior to the start of experiments. Please provide this information.

• The authors state that pregnant dams were ordered and delivered to their animal facility before giving birth to experimental animals, which is highly stressful for the animals. Stress in gestating mothers is known to affect social behaviors in animals. The authors should state their rationale for having the animals delivered or provide evidence that the transport stress does not meaningfully contribute to changes in the experimental animals’ social behavior.

• The behavior data was collected at 4 and 8 weeks after TBI, but the electrophysiology data was only collected at 7-8 weeks after TBI. The authors should write the discussion with this in mind since they cannot draw the same conclusions about the role of oxytocin in the social impairments observed at 4 weeks post-TBI without corresponding electrophysiology data. A rationale for only using the later timepoint would also be helpful for the reader.

• The authors do not appear to have used precision-targeting of the neurons in their whole-cell patch clamp recordings (e.g., fluorescent tagging) or post-hoc methods for assessing recorded neurons (i.e., neurobiotin and immunohistochemical staining). Rather, they seem to have recorded from any PFC layer 2/3 pyramidal neuron. If this is the case, they should specify a major limitation of their findings is that it was unknown whether the PFC neurons from which they recorded possessed oxytocin receptors.

Minor Comments:

• The figures are not numbered in the reviewer pdf, which should be corrected.

• None of the figures (except for figure 6) have legends, which makes it difficult to make a quick visual comparison between groups.

• The figures and graphs should be in a vector format. Trying to zoom in on them obscures data clarity since they are not vectorized.

• The authors switch between using 2/3 and II/III as notation for the pyramidal cells. This should be standardized unless it is referring to different groups of cells. If this is the case, this should be stated clearly.

• Figure 5c is difficult to interpret as is given here and initially appears to contradict the intended results. The graph and related descriptions should be changed to make it clear to the reader that discrimination indices above 0 are still indicative of animals that are making the correct choices in the behavioral tasks. Throughout the paper, the scale of the discrimination index graphs changes, which obscures this point.

• There were grammatical errors in the manuscript. The authors should carefully proofread the text.
